# Ecological Validity of Virtual Reality Daily Living Activities Screening for Early Dementia: Longitudinal Study

**DOI:** 10.2196/games.2778

**Published:** 2013-08-06

**Authors:** Ioannis Tarnanas, Winfried Schlee, Magda Tsolaki, René Müri, Urs Mosimann, Tobias Nef

**Affiliations:** ^1^Gerontechnology and Rehabilitation GroupUniversity of BernBernSwitzerland; ^2^University of UlmClinical & Biological Psychology, Institute of Psychology & EducationUlmGermany; ^3^Aristotle University of Thessaloniki3rd Neurological ClinicThessalonikiGreece; ^4^Division of Cognitive and Restorative NeurologyDepartments of Neurology and Clinical Research, InselspitalBern University Hospital and University of BernBernSwitzerland; ^5^Department of Old Age PsychiatryUniversity Hospital of Psychiatry and University of BernBernSwitzerland; ^6^ARTORG Center for Biomedical Engineering ResearchUniversity of BernBernSwitzerland

## Abstract

**Background:**

Dementia is a multifaceted disorder that impairs cognitive functions, such as memory, language, and executive functions necessary to plan, organize, and prioritize tasks required for goal-directed behaviors. In most cases, individuals with dementia experience difficulties interacting with physical and social environments. The purpose of this study was to establish ecological validity and initial construct validity of a fire evacuation Virtual Reality Day-Out Task (VR-DOT) environment based on performance profiles as a screening tool for early dementia.

**Objective:**

The objectives were (1) to examine the relationships among the performances of 3 groups of participants in the VR-DOT and traditional neuropsychological tests employed to assess executive functions, and (2) to compare the performance of participants with mild Alzheimer’s-type dementia (AD) to those with amnestic single-domain mild cognitive impairment (MCI) and healthy controls in the VR-DOT and traditional neuropsychological tests used to assess executive functions. We hypothesized that the 2 cognitively impaired groups would have distinct performance profiles and show significantly impaired independent functioning in ADL compared to the healthy controls.

**Methods:**

The study population included 3 groups: 72 healthy control elderly participants, 65 amnestic MCI participants, and 68 mild AD participants. A natural user interface framework based on a fire evacuation VR-DOT environment was used for assessing physical and cognitive abilities of seniors over 3 years. VR-DOT focuses on the subtle errors and patterns in performing everyday activities and has the advantage of not depending on a subjective rating of an individual person. We further assessed functional capacity by both neuropsychological tests (including measures of attention, memory, working memory, executive functions, language, and depression). We also evaluated performance in finger tapping, grip strength, stride length, gait speed, and chair stands separately and while performing VR-DOTs in order to correlate performance in these measures with VR-DOTs because performance while navigating a virtual environment is a valid and reliable indicator of cognitive decline in elderly persons.

**Results:**

The mild AD group was more impaired than the amnestic MCI group, and both were more impaired than healthy controls. The novel VR-DOT functional index correlated strongly with standard cognitive and functional measurements, such as mini-mental state examination (MMSE; rho=0.26, *P*=.01) and Bristol Activities of Daily Living (ADL) scale scores (rho=0.32, *P*=.001).

**Conclusions:**

Functional impairment is a defining characteristic of predementia and is partly dependent on the degree of cognitive impairment. The novel virtual reality measures of functional ability seem more sensitive to functional impairment than qualitative measures in predementia, thus accurately differentiating from healthy controls. We conclude that VR-DOT is an effective tool for discriminating predementia and mild AD from controls by detecting differences in terms of errors, omissions, and perseverations while measuring ADL functional ability.

## Introduction

A decade ago, Chaytor and Schmitter-Edgecombe [1] reviewed the ecological validity of neuropsychological tests by evaluating their efficacy in measuring everyday cognitive skills. They identified 6 studies that explored the issue of ecological validity of executive functioning tests. The studies differed in terms of the specific tests used, although both traditional (veridicality) and verisimilitude tests were employed. Veridicality refers to the extent to which results of an assessment instrument are related to scores on other tests that predict the performance of real-world tasks [2]. By contrast, verisimilitude refers to the similarity between the task demands of the test and the demands imposed in the everyday environment.

Their findings indicated that executive tests were not significantly correlated with self-reported measures, but all studies reviewed revealed significant associations between executive tests (traditional and verisimilitude) and everyday abilities as measured by clinician ratings and informants’ (eg, relatives’) questionnaires. To date, commentaries on ecological validity have primarily emphasized the increased consideration of this concept in assessments of neurologically impaired individuals, particularly in rehabilitative and forensic contexts. However, there are instances in which patients perform normally on traditional executive tests, yet clearly have executive impairments in their daily lives [3].

Virtual environments (VEs) have numerous features that make them attractive for assessment and rehabilitation purposes. In contrast to traditional executive test measures, VEs actively engage participants by allowing them to be involved in a task while at the same time being less focused on the fact that they are being tested [4,5]. More recently, researchers have used virtual reality (VR) systems for detailed response measurement and analysis to examine specific behaviors characteristic of patients with executive dysfunction or people with intellectual disabilities [6]. Klinger and colleagues [7] examined planning deficits in patients with Parkinson’s disease compared to age-matched controls in a virtual supermarket. The researchers described the patients’ paths through the supermarket as characterized by numerous stops, turns, and hesitancies as compared to the paths of controls. Zhang and colleagues [8] used a virtual kitchen to assess selected cognitive functions of traumatic brain injury patients compared to normal volunteers.

Task transparency and relevant functional tasks, such as finding one’s way through a VE or remembering groceries for preparing a breakfast in a virtual kitchen, are examples in which ecological validity can be described as enhanced when compared to abstract traditional assessments of cognitive functions. A variety of VEs have already been developed to enhance functional assessment and rehabilitation, including virtual cities [9,10], school classrooms [11], and supermarkets [9,12]. As outlined previously, ecological validity can be seen as a key component for assessing cognitive skills that are relevant for functional tasks in real-world contexts [13]. The results of such studies suggest that the use of VEs is valuable in enhancing our ability to assess the functional behaviors of individuals with executive dysfunction in activities of daily living.

Activities of daily living (ADL) can be classified into basic activities of daily living (BADL) and instrumental activities of daily living (IADL) [14]. BADL is composed of more basic self-care behaviors, such as ambulating, dressing, grooming, bathing, feeding, and toileting. By contrast, IADL facilitates independent living through behaviors such as transportation, telephone use, meal preparation, medication management, financial management, housekeeping, laundry, and shopping. IADL questionnaires play a vital role in assessing functional abilities and evaluating the impact of cognitive impairment on everyday activities in older adults [15].

IADL independence is one of the defining features that characterize normal aging from mild cognitive impairment (MCI) and dementia. As part of the diagnostic criteria for MCI, an individual must be classified as independent for BADL, but can have minimal disturbance in IADL [16,17]. Since the early descriptions of MCI [16], there has been increasing interest in its clinical characterization and prognosis [18,19]. In previous reports [20], people with MCI exhibited poorer cognitive functioning than healthy controls, but were not as impaired as patients with dementia were.

Prognostic studies have stressed the necessity of this nosological entity as a risk, or prodromal state for dementia, because of the high rate of conversion of MCI to dementia (10%-15% of patients who meet the criteria of amnestic MCI develop Alzheimer-type dementia per year, up to 80% at 5-year follow-up) [21,22,18]. In Europe, approximately 17% of the senior population who have not been diagnosed with dementia meet the current criteria for MCI [23] and MCI prevalence increases with age [24].

Characterizing impairment using the IADL questionnaire has been criticized for several reasons. First, no objective standard exists as to the practical or theoretical definition of minimal functional impairment in predementia [16,17]. For example, does functional disturbance entail perceptible impairment on a few IADL tasks, such as shopping and meal preparation? Or is it better understood as some problems across many commonly assessed IADL tasks? Clinical judgment is called for by the expert panel that created these standards [25,26], but the general clinician or researcher is without much guidance regarding how to assess IADL impairment in predementia patients. Several options exist, including performance-based tasks and questionnaires or interviews (with and without informant reports). However, different methods of assessing functional abilities require different estimates of IADL independence [27]; each method has advantages and disadvantages.

Recently, it became clear that IADL, versus BADL, is a better diagnostic instrument for predementia [28-31]. Although these studies were carried out in different countries and used different instruments to assess impairments in ADL, they all indicate that people who meet the criteria described for predementia show some functional impairment in activities of daily living. In the clinical setting, rate of change in complex ADL performance may be more useful than a cross-sectional measurement, which could misclassify individuals into a nonimpaired category in activities of daily living [32]. Furthermore, the rate of change is a parameter that could be manipulated by designing naturalistic VEs or serious games that can train the higher cognitive functions.

We designed a fire evacuation Virtual Reality Day-Out Task (VR-DOT) environment to (1) determine what kind of real-time cognitive and psychomotor performance and errors are associated with functional impairment in activities of daily living, (2) identify the patterns and cutoff values of the these cognitive and psychomotor profiles as independent predictors of functional impairment in healthy elderly participants, single-domain amnestic MCI patients, and patients with mild Alzheimer-type dementia (AD), and (3) controlling for baseline performance, objectively measure performance change over 2 to 3 years.

We hypothesized that with VR-DOT (1) dementia and MCI patients will show significantly impaired independent functioning in ADL and distinct performance profiles, (2) among patients with dementia or MCI, such impairment will be associated with the degree of cognitive impairment and cognitive neurophysiological measures, whereas impaired functioning will be only associated with sociodemographic and anxiety/depression symptoms in healthy controls because subclinical levels of cognitive impairment and depression have been associated with IADL impairment in mentally healthy participants [33], and (3) in mild AD and MCI patients, the rate of change in individual performance in VR-DOT measures could predict the cognitive decline over 2 to 3 years.

The main objective in developing the VR-DOT was to improve the ecological validity of executive function measures by using a verisimilitude approach. We also proposed a framework to objectively assess the functional impairment of elderly people through an ecological and clinical longitudinal experiment using VR-DOT. Our motivation was to correlate this new instrument (VR-DOT) with normal cognitive neuropsychological measures and recent psychomotor discoveries regarding psychomotor velocity change and cognitive decline to see if the VR-DOT offers better sensitivity and specificity in assessing and predicting cognitive decline using only a virtual environment.

## Methods

### Virtual Reality Test Setup

#### Overview

**Figure 1:**
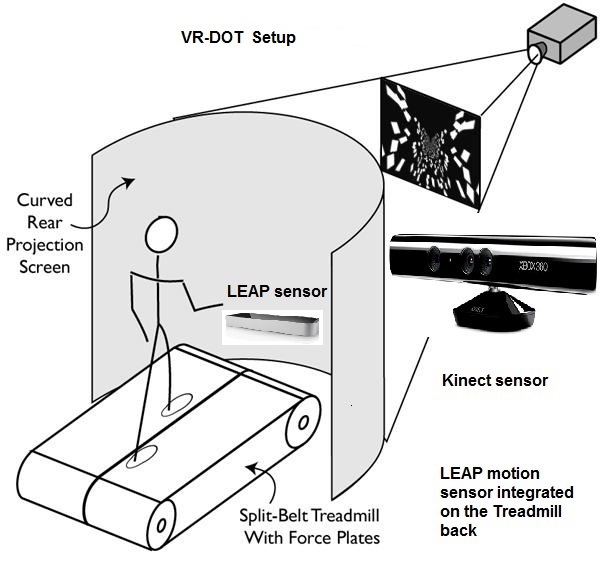
Virtual reality day-out task (VR-DOT) participant setup.

The VR hardware consisted of a Pentium-based computer with 4 MB RAM, Intel Quad Core processor, and NVIDIA graphic cards with 512 MB memory. Other sensors used were a LEAP motion sensor (Leap Motion Inc, San Francisco, CA, USA) and a Kinect camera (Microsoft Corp, Seattle, WA, USA). The LEAP motion sensor is still not commercially available at the time of this writing, but we were selected by their development team to use the hardware for our experiments (Figure 1).

#### Software Components

Modeling was done using Maya software (Autodesk Inc, San Rafael, CA, USA) to create models and scenes. Then, the scenes were exported to Virtools, a 3D authoring tool (Dassault Systèmes, Inc, Vélizy-Villacoublay Cedex, France) that handled all programming including interactivity, setting, and configuration. Microsoft Kinect software development kit (SDK) (Microsoft Corp, Seattle, WA, USA) was used to analyze gestures and movements and a user interface (UI) system was developed using Microsoft Kinect SDK and precommercial Alterniity algorithms developed by Ioannis et al [34].

#### The Naturalistic Setting of Executive Function in Virtual Reality Activities of Daily Living

**Figure 2:**
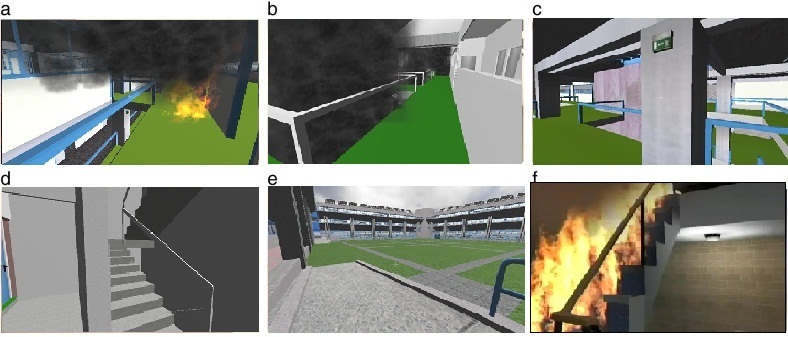
Sample sequential virtual reality day-out task (VR-DOT) screenshots, showing different tasks and viewpoints.

Virtual reality activities of daily living (VR-ADL) consists of 2 modules: the VR-DOT and VR basic instrumental activities of daily living (VR-IADLs). The VR-DOT is a complex task breakdown and then a rehearsal exercise of a fire evacuation drill consisting of 6 different scenarios of increasing difficulty. We chose to examine the VR-DOT virtual fire evacuation drill in this study (Figure 2), based on the literature indicating that activities of daily living requiring complex reasoning are sensitive to cognitive and functional impairment [35]. User tracking was performed by a flexible action and articulated skeleton toolkit (FAAST; University of Southern California, CA, USA), a middleware to facilitate integration of full-body control with games and VR applications, using either OpenNI or the Microsoft Kinect for Windows skeleton tracking software. FAAST includes a custom virtual reality peripheral network (VRPN) server to stream up to 4 user skeletons over a network, allowing VR applications to read the skeletal joints as trackers using any VRPN client. Additionally, the toolkit can also emulate keyboard input triggered by body posture and specific gestures. This allows the user to add custom body-based control mechanisms to existing off-the-shelf games that do not provide official support for depth sensors.

More specifically, the VR-DOT module is a naturalistic task that requires multitasking in a fire evacuation drill setting with 6 different simulated fire situations (from easy to more difficult) taking place at a virtual apartment block with 3 levels and 5 apartments per level. It is used to examine prospective memory as well as reasoning in a complex emergency routine in which older adults prioritize, organize, initiate, and complete a number of subroutines to evacuate safely from an apartment level (second floor) to the ground area (eg, determine and gather information on the size of the fire, avoid smoke). Previous research shows that motion tracking while navigating a virtual environment is a valid and reliable indicator of cognitive decline in elderly persons. (ie, [36]).

### Functional and Psychomotor Rate of Change

A measurement of the rate of change of functional impairment was computed from all information collected from the LEAP motion and the Microsoft Kinect camera sensor inside the VR-DOT. Simple performance-based functional impairment measures have been used previously, but not with data collected from motion sensors [37]. At baseline, a simple quantitative ratio of efficacy was computed by dividing the total time (in sec) spent by the participant performing the listed activities by the total time spent in VR-DOT (efficacy ratio). Then, 4 activity parameters with a high likelihood of corresponding to functional decline were collected: (1) omission of 1 of the activities (*k*_1_), (2) repetition of the same activity (*k*_2_), (3) incorrect order in performing the activities (*k*_3_), and (4) number of attempts before completing a given activity (*k*_4_). The first quantitative ratio of efficacy was then adjusted by these parameters. This led to a functional impairment score according to the formula presented in Multimedia Appendix 1.

To determine values of the model parameter set (k_1_, k_2_, k_3_, k_4_), we ran a pilot with healthy participants (n=25; mean age 73.7 years, SD 4.0), amnestic single-domain MCI patients (n=26; mean age 74.2 years, SD 2.0), and patients with mild AD (n=24; mean age 76.7 years, SD 3.0). Second, multiple-model parameter sets (k_1_, k_2_, k_3_, k_4_) to produce a good fit were selected if their associated scores were both strongly and positively correlated with the mini-mental state examination (MMSE) scores, as well as being strongly and negatively correlated with IADL scores using a nonparametric Spearman correlation coefficient as the criterion distance of good fit. For our analyses, the final functional impairment score (k_1_, k_2_, k_3_, k_4_) was calibrated using the combination of the mean of the parameters, which was selected as the model parameter set during the second step of the fitting procedure.

### Procedure

#### Participants

A total of 405 elderly people were screened during 2010 in 2 Alzheimer Hellas, Non-Government Organization (NGO) day clinics of the Papanikolaou University Hospital in Thessaloniki, Greece. Ethics approval was obtained from the Papanikolaou University Hospital Ethics Committee. Inclusion criteria were age older than 60 years, meeting the diagnostic criteria for MCI as defined in Petersen et al [18], living in the community, and providing informed consent approved by the Ethics Committee. Exclusion criteria were living in an assisted-living residence, cognitive functioning suggesting a possible diagnosis of dementia (see subsequent description), previous diagnosis of dementia, other psychiatric disorder according to the *Diagnostic and Statistical Manual of Mental Disorders* (Fourth Edition, Text Revision; *DSM-IV-TR*) at the time of recruitment, presenting a moderate or higher degree of fear or dislike of computers (technophobia), presenting a moderate or higher degree of disability because of other conditions than MCI, and severe language impairments that would compromise active participation.

The baseline psychomotor evaluation inside VR-DOT consisted of a number of simple and complex measures addressing the participant’s ability to understand and perform with accuracy specific physical performance tasks. These tasks/metrics were:

##### Grip Strength

Forearm muscle strength was measured in kilograms by a hand-held Jamar A dynamometer. For this analysis, we used the best of 3 attempts in the dominant hand.

##### Timed Walk on the Treadmill

The time (to 0.1 s) required for a participant to walk a 4.6-m course at his or her usual pace after starting from a standstill was recorded by stopwatch. We converted the results to meters per second.

##### Number of Steps to Walk Course on the Treadmill

A technician recorded the number of steps required to walk the 4.6-m course. Hereafter, we refer to stride length, which is derived by dividing the distance walked by the number of steps.

##### Finger-Tapping Test

Using their dominant hand, participants tapped in midair, just above the LEAP motion sensor, with the index finger as fast as they could for 15 seconds.

After applying the inclusion/exclusion criteria, 232 participants were included in this study. The participants were measured each year of the 3-year study period. Dropouts after baseline were 27 (11.6%); hence, 205 participants completed all measurements and their data are included in this study (N=205; male=88, female=117; mean age 72.73 years, SD 6.89; mean education 12.53 years, SD 3.20; mean baseline MMSE 24.75, SD 2.18).

#### Neuropsychological Assessment

Cognitive assessment was performed by means of a neuropsychological test battery designed to comprehensively evaluate attention, working memory, memory, executive functioning, and language. In addition to the cognitive assessment, all groups were also assessed for depression with the geriatric depression scale (GDS) [38]. We also chose the Digit Symbol (DSym), Functional Activities Questionnaire (FAQ), Neuropsychiatric Inventory brief questionnaire form, Apathy item (NPI-Q Apathy), Neuropsychiatric Inventory brief questionnaire form, Depression item (NPI-Q Depression), Rey Auditory Verbal Learning Test T (RAVL), Trailmaking Test A (TMT-A), Trailmaking Test B (TMT-B), Trailmaking Test B minus Trailmaking Test A (TMT-B-A), the Bristol ADL scale, and the short form of the Blessed ADL scale for this study because they were evaluated and validated for the Greek population [39]. The original Bristol and short-form Blessed scales consist of 20 and 11 items, respectively.

#### Statistical Analysis

Performance results from the VR-DOT, gait velocity assessment, and neuropsychological tests were analyzed using multivariate analyses of variance (MANOVA) in mixed designs with group as the between-subject factor using linear mixed-effects models with random intercept and slope to estimate the annual rate of change between study years 1 and 3 for each performance measure of each participant [40,41]. Before this approach, we plotted numerous individual trajectories for the gait velocity performance variables by using robust splines to smooth the curves. The consistent linearity of the trajectory patterns justified the use of linear models. Gait speed and stride length were adjusted to a 50-cm knee-heel length and this adjustment was included in the models when it reached 10% significance. We also used multinomial Poisson log-linear models to estimate the relative risk (RR) of cognitive decline relative to efficacy, gait velocity, and neuropsychological assessment at year 2 and year 3 (2010-2013) for VR-DOT and receiver-operating curve (ROC) analysis was conducted on VR-DOT, MMSE, the RAVLT, and the Bristol and Blessed ADL scale scores.

Significant effects were further tested with post hoc tests that were corrected for multiple comparisons using Tukey’s Honestly Significant Difference (HSD) [42]. We used similar statistical models to estimate the RR of having significant VR-DOT difficulty or inability (relative to no or mild difficulty) and the RR of cognitive decline (relative to no or mild difficulty) at year 3 for upper extremity function. For a given performance measure, the first year 1 value and the third year 1-3 slope of change were treated as separate predictor variables. For each outcome, a separate regression model was run for each predictor performance variable, adjusting for age, gender, and the VR-DOT task of more difficulty with, or disability in, the outcome measure between years 1 and 3. Next, we simultaneously entered all predictor performance variables into a second set of models, adjusting for the same covariates. The component variables from each model were entered, in turn, into a stepwise backward regression for the respective outcomes, with a *P* value to enter the model set at <.10. This procedure yielded a set of simpler, more parsimonious final models. All statistical analyses were run using SPSS 19.0 statistical software (IBM Corp, Armonk, NY, USA).

## Results

Demographics and baseline scores for all groups are shown in Table 1.

After corrected with age, gender, and education status, our results showed that the VR-DOT functional index was correlated strongly with standard cognitive and functional measurements, such as MMSE (rho=0.26, *P*=.01) and Bristol ADL scores (rho=0.32, *P*=.001), thus accurately differentiating from healthy control participants (Table 2).

In the prediction models for individual performance measures (not shown), the VR-DOT and upper extremity function psychomotor performance (finger tapping, etc) at year 3 for the MCI and mild AD group, as well as the slopes of change, had a significance of *P*<.10. Compared with the control, weaker results of the MCI and mild AD independently predicted cognitive decline at year 3 in all 3 domains (VR-DOT, neuropsychological, and gait velocity assessment). The change slope for upper extremity function inside the VR-DOT was also associated with the outcome.

For functional independence, the healthy group showed better functional adjustment than the MCI and mild AD group according to VR-DOT total monitoring data. When the amnestic MCI group was examined using the VR-DOT total score, cognitive domain and gait velocity assessment showed a similarly impaired profile as cognitive functioning, after controlling for age, education, and GDS score. The mild AD patients showed a higher degree of functional impairment than both healthy controls and amnestic MCI patients in life activities, and participation subscales, respectively, and in the VR-DOT mobility domain. The total VR-DOT functional ability measures showed a consistent functional impairment of mild AD and amnestic MCI in comparison with healthy participants.

**Table 1:** Participant demographics and scores on cognitive tests for all participants, healthy controls, patients with amnestic-type mild cognitive impairment (aMCI), and patients with mild Alzheimer-type dementia (AD).

Group		All participants N=205	Controls n=72	aMCI n=65	Mild AD n=68
Age, mean (SD)		72.73 (6.8)	72.63 (5.06)	72.78 (6.21)	72.58 (6.21)
**Sex, n (%)**					
	Male	88 (43%)	25 (38%)	30 (43%)	33 (46%)
	Female	117 (57%)	37 (62%)	40 (57%)	40 (54%)
Education, mean (SD)		15.6 (3.0)	16.1 (2.9)	15.7 (3.0)	14.6 (3.2)
**Test,** ^a^ **mean (SD)**					
	MMSE	24.75 (2.18)	29.1 (1.0)	26.1 (1.8)	23.4 (2.0)
	RAVLT delayed recall	3.7 (4.0)	7.4 (3.7)	2.9 (3.3)	0.7 (1.6)
	GDS	1.4 (1.4)	0.8 (1.1)	1.6 (1.4)	1.6 (1.4)
	NPI-Q depression	0.2 (0.5)	0.1 (0.3)	0.2 (0.5)	0.4 (0.6)
	NPI-Q apathy	0.2 (0.6)	0.01 (0.1)	0.2 (0.5)	0.5 (0.8)
	FAQ	4.8 (6.4)	0.1 (0.6)	3.8 (4.4)	12.7 (6.7)
	TMT-A	46.6 (25.5)	36.3 (13.0)	44.2 (21.7)	64.8 (34.5)
	TMT-B	134.5 (80.2)	89.3 (44.3)	130.8 (73.2)	200.5 (86.6)
	TMT-B–A	88.0 (66.9)	53.0 (38.8)	86.6 (63.1)	135.8 (74.3)
	Bristol ADL scores	6.88 (0.56)	4.46 (0.5)	5.59 (0.9)	10.59 (0.9)
	Blessed ADL impairment score	2.87 (0.26)	1.85 (0.27)	2.38 (0.56)	4.38 (0.56)
	Geriatric depression scale	5.19 (5.0)	4.59 (4.1)	5.49 (5.76)	5.29 (4.45)
	Digit Symbol	37.4 (12.9)	45.8 (10.2)	37.0 (11.1)	27.6 (12.5)
**Gait speed (m/s), mean (SD)**					
	Combined	0.91 (0.22)	0.96 (0.23)	0.91 (0.24)	0.86 (0.20)
	Women	0.85 (0.14)	0.94 (0.24)	0.84 (0.04)	0.77 (0.14)
	Men	0.98 (0.13)	1.00 (0.21)	1.01 (0.03)	0.95 (0.04)
**Tapping speed dominant (taps/second), mean (SD)**					
	Combined	3.79 (0.8)	3.87 (0.8)	3.77 (0.81)	3.74 (0.8)
	Women	3.48 (0.78)	3.53 (0.71)	3.49 (0.84)	3.43 (0.77)
	Men	4.23 (0.75)	4.29 (0.77)	4.21 (0.73)	4.19 (0.75)
**Tapping speed non-dominant (taps/second), mean (SD)**					
	Combined	3.60 (0.67)	3.63 (0.64)	3.61 (0.71)	3.58 (0.7)
	Women	3.73 (0.59)	3.41 (0.53)	3.38 (0.64)	3.33 (0.61)
	Men	3.92 (0.63)	3.91 (0.65)	3.96 (0.63)	3.90 (0.62)

^a^MMSE: mini-mental state examination, RAVLT: Rey Auditory Verbal Learning Test, GDS: Geriatric Depression Scale, NPI-Q Depression: Neuropsychiatric Inventory brief questionnaire form, Depression item, NPI-Q Apathy: Neuropsychiatric Inventory brief questionnaire form, Apathy item, FAQ: Functional Activities Questionnaire, TMT-A: Trailmaking Test ), TMT-B: Trailmaking Test B, TMT-B–A: Trailmaking Test B minus Trailmaking Test A, ADL: Activities of Daily Living.

**Table 2:** The correlation matrix between Virtual Reality Day-Out Task (VR-DOT) functional index, mini-mental state examination (MMSE), and Bristol Activities of Daily Living (ADL) when controlling for age, gender, and education status.

Test	VR-DOT		MMSE		Bristol ADL
	rho	*P* value	rho	*P* value	rho
VR-DOT	1				
MMSE	0.26	.01	1		
Bristol ADL	0.32	.01	0.43	.01	1

### Predictors of Functional Status (Regression Analyses)

When the entire sample was analyzed together, attention, psychomotor, and memory summary scores explained a total variance of 8.2% and 0.8% of the VR-DOT. When depression and age were entered in both former models, the VR-DOT score was predicted by depression symptoms as measured by the GDS (19.2%) only in the healthy group. By contrast, VR-DOT total score was only predicted by psychomotor and executive functions (8.1%) among mild AD and amnestic MCI patients. For the amnestic MCI group, VR-DOT was predicted by executive functions and psychomotor profile only, with a total variance explained of 17.3% for amnestic MCI. VR-DOT score was predicted only by executive function and psychomotor profiles in amnestic MCI patients and by executive function, psychomotor profiles, and GDS scores in mild AD. When a ROC analysis was carried out on the Bristol and Blessed ADL scales, they explained 9.1% variance of VR-DOT total profiles.

ROC analysis was conducted on VR-DOT, MMSE, RAVLT, and Bristol and Blessed ADL scale scores obtained from the amnestic MCI and mild AD groups and the sensitivity, specificity, and cutoff values of both the scales were determined (Table 3). The optimal cutoff score of the Bristol scale was 20 in differentiating amnestic MCI from mild AD with a sensitivity of 100% and specificity of 74.2%, and area under the curve (AUC) of 0.883 (95% CI 0.781-0.975). The optimal cutoff score of the modified Blessed scale was 10.5 in differentiating amnestic MDI from mild AD with a sensitivity of 100%, specificity of 71%, and AUC 0.872 (95% CI 0.791-0.977). Post hoc analysis revealed that among the 3 groups, the mild AD group had the lowest scores in ADL, episodic memory, and VR-DOT (*P*<.001).

**Figure 3:**
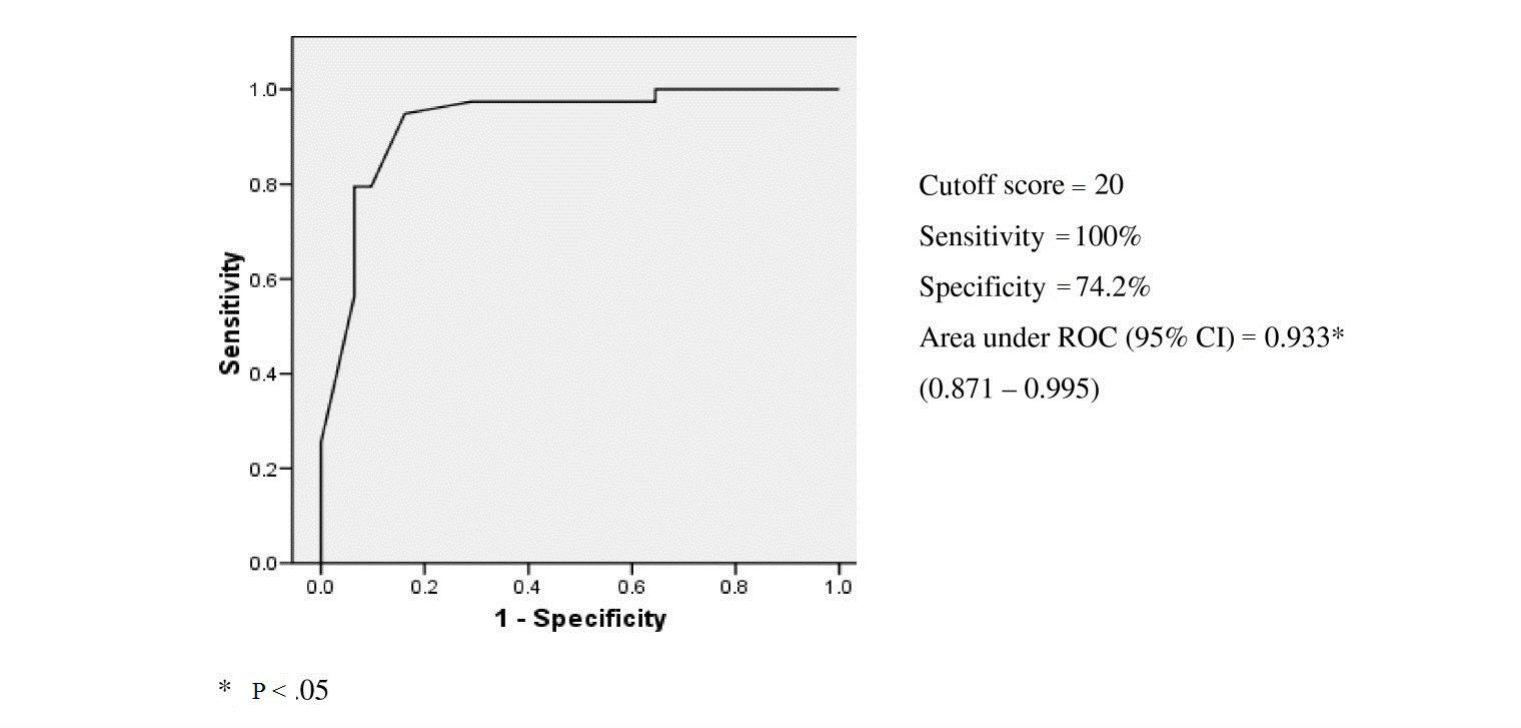
Receiver-operating curve (ROC) for the Virtual Reality Day-Out Task (VR-DOT) total score when discriminating among nondemented (healthy controls), amnestic mild cognitive impairment (aMCI), and patients with mild Alzheimer-type dementia (mild AD).

The AUC indicates that VR-DOT was the most powerful of all tests in discriminating normal controls from the MCI groups, reaching optimal results with a cutoff point of 20 (97% sensitivity, 100% specificity, 100% positive predictive values, and 96% negative predictive value). Figure 3 shows the ROC of the normal control and MCI groups for VR-DOT total score.

#### Exploratory Prediction of Conversion to Alzheimer Disease (VR-DOT Performance Rate of Change)

**Figure 4:**
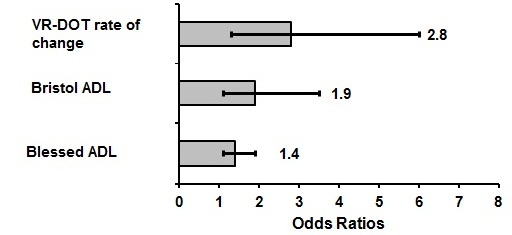
Odds ratios from exploratory individual regression analyses using VR-DOT, Bristol, and Blessed ADL scale scores rate of change as predictors for conversion from mild cognitive impairment to Alzheimer disease (bars represent 95% CI).

According to the results, the task that better differentiated among healthy controls, amnestic MCI, and mild AD participants at baseline, year 2, and year 3 follow-up, was the VR-DOT performance score (efficacy ratio). The VR-DOT and Bristol and Blessed ADL scale scores were included as predictor variables in a series of exploratory independent regression analyses. Figure 4 shows the individual predictive power of the 3 test variables of interest (VR-DOT, Bristol, and Blessed ADL scale scores), ranked in ascending order according to the magnitude of their odds ratios. The VR fire evacuation performance score rate of change (VR-DOT REff) emerged as the best predictor of conversion to AD in this sample (VR-DOT; *P*=.008; OR 2.8, 95% CI 1.3-6.0; Nagelkerke *R* ^*2*^=0.564), with the regression model correctly classifying 88% of participants. This was followed by Bristol ADL (*P*=.03; OR 1.9, 95% CI 1.1-3.5; Nagelkerke *R* ^*2*^= 0.563), and the Blessed ADL (*P*=.01; OR 1.4, 95% CI 1.1-1.9; Nagelkerke *R* ^*2*^=0.466).

The resulting regression model revealed that the VR-DOT performance score threshold variable was a significant predictor of conversion to AD in the regression equation (beta=–1.092, *P*=.01) with OR 3.0 (95% CI 1.3-7.0). Using a cutoff score of less than 20 on the VR-DOT subscale achieved a sensitivity of 100% and a specificity of 94%.

**Table 3:** Area under the curve (AUC) for standard neuropsychological test scores and Virtual Reality Day-Out Task (VR-DOT) for healthy controls versus patients with amnestic-type mild cognitive impairment (aMCI) and patients with aMCI versus patients with mild Alzheimer-type dementia (AD).

Test^a^	Healthy control vs aMCI		aMCI vs mild AD	
	AUC (95% CI)	*P* value	AUC (95% CI)	*P* value
MMSE	0.79 (0.68, 0.91)	<.001	0.99 (0.97, 1.00)	<.001
Bristol scores ADL	0.75 (0.62, 0.88)	<.001	0.88 (0.78, 0.97)	<.001
Blessed score ADL	0.77 (0.64, 0.89)	.002	0.87 (0.79, 0.98)	.02
RAVLT delayed recall	0.82 (0.77, 0.93)	.001	0.88 (0.79, 0.98)	<.001
DOT-VR	0.96 (0.88, 0.99)	<.001	0.95 (0.88, 1.00)	<.001

^a^MMSE: Mini-Mental State Examination, ADL: Activities of Daily Living, RAVLT: Rey Auditory Verbal Learning Test.

## Discussion

There is still debate as to the utility of MCI as a diagnostic category. Many older people report subjective cognitive complaints in the absence of objective impairment [43] and not all such complaints are predictive of dementia [44]. MCI may be viewed as being on a continuum from normal aging to dementia and the present data show a large overlap between groups that coheres with this view [45]. In that context, only a few studies have systematically examined the rate of change in complex ADL performance as a predictor of cognitive decline.

Our results show that functional impairment is a defining feature of both amnestic MCI and mild AD, and that the impairment showed by amnestic MCI patients is partially dependent on the degree of their cognitive impairment. Furthermore, a virtual reality quantitative performance measure of functional ability (VR-DOT) showed adequate psychometric properties (ie, discriminant power) to contribute to a predementia diagnosis. In addition, functional measures based on quantitative rates of the number and quality of ADL performed seem to be more sensitive to identifying functional impairment in predementia than those based on a subjective judgment of disability.

As a result of this paradigm shift, and in light of previous and the present results, it would be very helpful for clinicians, caregivers, and health-system managers if MCI definitions included an objective measure of impairment of functional abilities as a clinical feature inherent to MCI. We found that VR-DOT has greater sensitivity and specificity, as well as having both positive and negative predictive values compared to other screening tests in discriminating amnestic MCI and mild AD from normal aging. In summary, these and previous results emphasize the presence of qualitative and quantitative functional impairments of both basic and complex ADL in predementia as a logical consequence of cognitive impairment. Although dementia is characterized by a more severe degree of disability than predementia, the World Health Organization (WHO) International Classification of Functioning, Disability and Health (ICF) conceptualization of disability would include predementia as a disabling condition, although to a lesser degree. The need for a better definition of disability as a diagnostic criterion (putatively by shifting from a categorical notion of able/disabled to a more spectrum/gradual approach) to discriminate predementia from dementia must not conceal the fact that dementia patients have their own health/functional assistance needs.

Moreover, given the moderately good psychometric properties demonstrated in our study of the VR-ADL in discriminating healthy from predementia and mild dementia patients, assessing real-time functional ability would improve the identification of predementia patients, and the use of objective, VR qualitative and/or quantitative impairment of functional abilities as a diagnostic criterion should be further explored. Goldberg et al [26] found that a sensitive performance-based measure they developed (the University of California, San Diego Performance-Based Skills Assessment; UPSA) had a remarkably good discriminant power to distinguish healthy participants from amnestic MCI participants (AUC 0.84), and to distinguish amnestic MCI patients from patients with AD (AUC 0.88). Hence, the inclusion of functional competence measures seems convenient for the screening and early identification of neurodegenerative processes characterized by cognitive impairment.

The rates of change in complex everyday activities, easily determined in longitudinal practice settings, provide important prognostic information for late-life disability and death that are independent of the predictive value of a performance measurement obtained at a single point in time, which could be inaccurate because of recent injury or illness. By predicting decline in ADL and IADL, upper extremity functionality, and more generalized daily activities, longitudinal views capture broader deteriorations in function within an individual, suggesting a shared causal pathway.

Our study has limitations. Although we used a population-based cohort, the exclusion from the analyses of participants with technophobia [46] may have introduced bias and reduced the generalizability of the results. Although we only observed linear patterns in the many performance trajectories that we plotted, some individual trajectories could have been nonlinear causing inaccurate estimates of annual performance change. Our statistical models contained a limited number of covariates. Although the addition of comorbid conditions to the models did not significantly alter the results, we may have omitted important confounders.

The present research described the ecological validity of verisimilitude and traditional activities of daily living measures and the characterization of various subcomponents of the executive function system. The unique contribution of this study was in the development and empirical study of a novel VR environment (VR-DOT) that was less structured and that more closely resembled actual everyday errands than existing questionnaires. This research demonstrated that tests of verisimilitude may be better predictors of real-world behaviors than many of the most commonly employed traditional executive function tests.

Our approach with VR-DOT is part of a general effort to manifest marked impairment in cognitive performance, particularly executive functions during everyday activities by means of VR (VR-ADL). Studies directly investigating ADL have found mild and tardive impairment in MCI, and a relation with certain executive functions, but the targeted ADL were very simple tasks, such as memorizing a telephone number or walking a few meters, and have always been strictly limited to the accuracy domain, excluding any performance or a rate-of-change factor. The purpose of VR-DOT was (1) to investigate performance, in an experimentally controlled manner, on a complex ADL (planning and evacuating a fire under time pressure) that is more indicative of the true quality of life of senior citizens, and (2) to scrutinize its cognitive structure as a diagnostic instrument, which can screen functional impairments at a very early phase of AD. With regard to real-life ADL, this investigation presents the advantage and innovation of a VR quantitative scoring grid of a very complex set of sequential activities under demanding time constraints.

This study found that VR-DOT is comparatively better in detecting amnestic MCI from normal aging individuals. From quantitative and qualitative data extracted from VR-DOT, a functional index was computed, validated, and compared with current clinical rating scales. Results of this pilot study are promising and must be substantiated with a larger sample and in another assessment setting to evaluate its reproducibility. Verisimilitude instruments, such as VR-DOT, can potentially play valuable roles in both executive function assessment and intervention and, consequently, may help place clinical neuropsychology on firmer scientific ground. Researchers and clinicians have the responsibility and opportunity to design, test, and implement effective therapeutic strategies to improve, or at least preserve, functional and cognitive functioning in predementia.

For these purpose, it is assumed that the visual quality and realism of the VEs are of central importance for patients to recognize and acknowledge the relevance of the task and context at hand. Essential characteristics of virtual scenarios and tasks (ie, transparency, believability, plausibility, and relevance) are summarized under the term “realism” to describe that the patient can recognize the employed tasks and scenarios and refer to them based on past experiences. VR-ADLs capture the patient’s interest and improve long-term motivation to use the virtual tasks at high frequencies. Transparency and realism in a broader sense can relate to plausibility and place illusions that are described by Slater [47]. Plausibility illusion refers to the fact that the user believes the virtual scenario is actually occurring. It is caused by events and the scenario relating directly to the user (eg, the virtual character talking to the user). Place illusion refers to the sensation that the user is actually situated in the displayed location and is described in relation to sensorimotor contingencies of the VR system (eg, user interaction, tracking, and multimodal user feedback). VR-ADL, task transparency, and relevant virtual scenarios are believed to contribute to the described illusions that virtual events and locations are actually relevant to the user and engaging for cognitive rehabilitation. For example, a cognitive task embedded in a user-relevant scenario directly relates to the therapy goal of the patient and represents a desired outcome of the patient’s rehabilitation (eg, a virtual kitchen with cooking tasks relates to the scenario that the patient aims to engage in independently at home).

The VR system used here is portable and can be manipulated to simulate different environments and different navigation demands (cognitive, motor, visual), easily allowing the creation of an ecologically valid study and testing in a variety of clinical and research settings.

In conclusion, relative to age-matched controls, VR-ADL exercises outperform the clinical predictive validity of traditional assessments as an indicator of real-world difficulties in IADLs. This result is very promising, but we will need advanced imaging techniques, such as amyloid-positron emission testing or functional magnetic resonance imaging, to study this relationship and perform a longitudinal study that would correlate our results with neuroimaging data as well.

## Multimedia Appendix 1

Efficacy ratio and functional impairment score formula.

## Abbreviations

AD: Alzheimer disease
ADL: Activities of Daily Living
AUC: area under the curve
BADL: basic activities of daily living
GDS: geriatric depression scale
IADL: instrumental activities of daily living
MANOVA: multivariate analyses of variance
MCI: mild cognitive impairment
MMSE: mini-mental state examination
RAVLT: Rey Auditory Verbal Learning Test
ROC: receiver-operating curve
RR: relative risk
UI: user interface
VE: virtual environment
VR: virtual reality
VR-DOT: Virtual Reality Day-Out Task
VRPN: virtual reality peripheral network
