# Supplementary figures and images for: Ecological Validity of Virtual Reality Daily Living Activities Screening for Early Dementia: Longitudinal Study
Source: JMIR Serious Games. 2013 Aug 6;1(1):e1. doi: 10.2196/games.2778 (PMC4307822; doi:10.2196/games.2778)

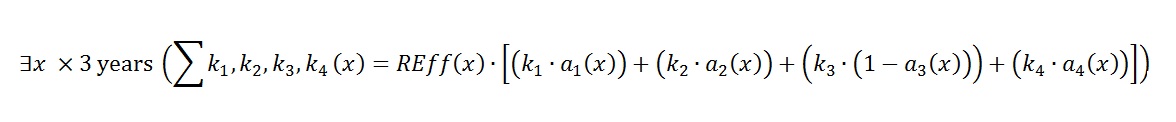

Supplement: Supplementary file 1 [file games_v15i8e156_app1.jpg]
